# A qualitative evaluation of the effectiveness of behaviour change techniques used in the Healthy Eating and Active Lifestyles for Diabetes (HEAL-D) intervention

**DOI:** 10.1186/s12889-025-21767-8

**Published:** 2025-02-11

**Authors:** A. P. Moore, C. Rivas, S. Harding, Louise M. Goff

**Affiliations:** 1 University College London, Gower Street, London, WC1E 6BT UK; 2 King’s College London, Franklin Wilkins Building, London, SE1 UK; 3 Diabetes Research Centre, Leicester General Hospital, University of Leicester, Gwendolen Road, Leicester, LE5 4PW UK

**Keywords:** Type 2 diabetes, Ethnicity, Behaviour change, Process evaluation, Self-management, Behaviour change, COM-B

## Abstract

**Background:**

Type 2 diabetes (T2D) is up to three times more common in people of Black African and Black Caribbean heritage living in the UK, compared to their White British counterparts. Structured education is the cornerstone of care but is less successful for people from minority ethnic groups. Healthy Eating and Active Lifestyles for Diabetes (HEAL-D) was developed to support diabetes self-management in people of Black African and Caribbean heritage living with T2D in the UK. The intervention was designed using COM-B/behaviour change wheel methodology to specify the theory of change. In a process evaluation study, we explored how the selected behaviour change techniques (BCTs) supported behaviour change in the intervention.

**Methods:**

Focus groups and interviews were conducted with participants who were randomised to receive the HEAL-D intervention in a feasibility trial. A topic guide directed discussions to explore experiences of HEAL-D, key learnings and impact, and behaviour change; the interviews gave the opportunity to probe further the focus group themes and areas requiring clarification. Sessions were audio-recorded and transcribed. Framework analysis was used to explore how the selected BCTs supported behaviour change in those attending HEAL-D.

**Results:**

Thirty-six participants took part in one or both activities (44% Black African, 50% Black Caribbean, 6% Mixed race; 61% female, 83% first-generation; mean age 59.5 years, SD 10.02). Participants reported increased physical activity, reduced carbohydrate portion size and engagement in weight monitoring behaviour. BCTs to increase social opportunity (*social comparison*,* social support*) and overcome motivational barriers (*credible sources* and *modelling*) were effective in addressing cultural barriers relating to diet, stigma and health beliefs. BCTs to develop capability (*demonstration*,* instruction*,* information on health consequences*) were effective because of the cultural salience of the developed components. Less impactful BCTs were *problem solving*, *graded tasks*, *goal setting*, and *feedback on outcomes*.

**Conclusions:**

BCTs in the HEAL-D intervention were effective in supporting behaviour change, particularly those promoting social opportunity, as normative cultural habits and beliefs can conflict with diabetes self-management guidance. In addition, lifestyle interventions should include opportunity for experiential learning alongside culturally salient information provision.

**Trial registration:**

number: NCT03531177, May 18th 2018.

**Supplementary Information:**

The online version contains supplementary material available at 10.1186/s12889-025-21767-8.

## Background

Type 2 diabetes (T2D) prevalence is up to three times higher in UK Black African and Black Caribbean adults compared to their White British counterparts [1]. It is predicted that by the age of 80, 40–50% of UK adults from Black ethnicities will have T2D [2], compared to 14% in the White British population [3]. Moreover, diagnosis occurs up to 10 years earlier than in White British, with poorer glycaemic control at diagnosis [4]. A range of issues contribute to this increased prevalence including socioeconomic, cultural, lifestyle and genetic factors [5]. People from Black minority ethnic groups are also less likely to complete annual T2D monitoring and are subject to prescribing disparities [6] and other structural barriers to equitable access [7]. Diabetes self-management is the cornerstone of T2D care and refers to the active participation of patients in the management of their condition [8]. It involves adherence to dietary and physical activity advice, engagement with weight control and following an appropriate medication regimen [8]. In the UK, adults living with T2D are offered self-management structured education to increase knowledge, skills and motivation to self-manage their condition. This evidence-based education is delivered in groups, in medical and community settings, and teaches participants about dietary control of blood glucose, the role of physical activity and how to use diabetes medications appropriately to effectively manage their own health. Attendance at structured education is associated with improved glycaemic control [9], but people from minority ethnicities are less likely to attend [10], and, for Black participants, attendance is less likely to be associated with improved outcomes [11].

A range of sociocultural factors affect diabetes self-management, including relationships with healthcare providers, community and cultural influences, socioeconomic circumstances and other environmental factors [12]. As day-to-day self-management happens primarily in the social rather than clinical environment, sociocultural factors, such as normative dietary patterns, explanatory models of health, spiritual beliefs, and taboo and stigma, all influence engagement and outcomes [13]. Clinical guidelines in the UK identify a need for diabetes care to be culturally appropriate for people from different ethnicities [14] and a robust body of evidence suggests that culturally tailored diabetes education can improve outcomes in both glycaemic control and knowledge [5, 15]. However, there have been limited interventions designed to support people of Black African or Caribbean heritage in the UK setting [5]; the Healthy Eating and Active Lifestyles for Diabetes (HEAL-D) intervention was designed to address this need.

HEAL-D is a culturally tailored diabetes self-management education and support programme, which was co-designed with Black African and Caribbean adults, with the objective of improving engagement with self-management guidance and supporting positive diabetes-related health behaviours [16, 17]. During the development of HEAL-D, people reported several barriers to optimal self-management including social pressures and personal desire to continue to enjoy traditional cultural foods, lack of empathy from or distrust of healthcare, traditional beliefs relating to body shape and a rejection of the relevance of BMI ranges for the Black body shape. Additionally, a lack of detailed understanding of diabetes-related physiology, cultural attitudes towards food preparation, sociocultural challenges around the pressures of caring roles, inflexibility of shift work and ‘zero hours’ contracts, and socioeconomic pressures were evident from the preliminary development work [18].

In developing the HEAL-D intervention, the COM-B framework and behaviour change wheel (BCW) [18, 19] were used to choose specific behaviour change techniques (BCTs) to aid behaviour change amongst the participants. The COM-B model conceptualises barriers and enablers to behaviour change related to an individual’s capability (knowledge and skills), opportunity (environmental and social factors) and motivation (beliefs and intentions) [19]. Based on the COM-B analysis, the BCW then supports the researcher to identify suitable intervention functions e.g. to educate or persuade, and then choose appropriate evidence-based BCTs. BCTs are theoretically informed strategies shown to target specific determinants of behaviour and to effect change in a defined manner. For example, the BCT *Instruction on how to perform the behaviour* is designed to educate by increasing knowledge and skill. The BCTs used in HEAL-D are listed in Table 1. Intervention components, designed to deliver the selected BCTs in HEAL-D, included games, videos, exercise classes and education sessions. The intervention was delivered face-to-face over 7 sessions, in community settings, as part of a feasibility trial from March 2018 to April 2019 [20, 21]. Details of the HEAL-D intervention [22] and the COM-B analysis and the identification and operationalisation of the BCTs have been previously described [18]. Exploring the mechanisms through which interventions bring about change is crucial to understanding how the effects of a specific intervention occur as well as how these effects might be replicated or improved upon in similar future interventions [20, 21]. As part of an embedded process evaluation within the HEAL-D feasibility trial, we aimed to understand how the HEAL-D intervention supported behaviour change, and participant engagement with the selected BCTs.

**Table 1:** Description of the HEAL-D intervention components to support each BCT identified. Items in brackets refer to the BCW intervention function and the mechanism of action of the BCT. (modified from Moore et al.,2019) [18]

BCT	Intervention component
**Information about health consequences** *(To educate developing knowledge and skills)*	The educational curriculum covered health consequences and benefits of various key lifestyle behaviours. A detailed file provided contained written information and activities to support each educational session. An animation video *“Diabetes explained”* explained the mechanisms of type 2 diabetes.
**Instruction on how to perform the behaviour** *(To educate developing knowledge and skills)*	The curriculum communicated health guidance clearly using culturally relevant examples.
**Demonstration** *(To educate developing knowledge and skills)*	Practical games, the weekly discussion tasks, a cooking session (with cooks in the family invited) and structured exercise sessions (including African dance music and dancing) provided guided demonstration. An exercise DVD using credible sources was provided for participants to follow at home.
**Graded tasks** *(To educate developing knowledge and skills)*	Physical activity sessions and targets were graded for ability to boost chances of success hence confidence and self-efficacy.
**Social support (unspecified)** *(Socially focused to Persuade and Enable)*	Social connectedness was fostered within the group by the discursive nature of the sessions and through shared engagement in activities and structured exercise sessions
**Social comparison** *(Socially focused to Persuade and Enable)*	The *‘task card’* homework activities gave participants opportunity to try the lifestyle targets and come back to discuss with the group and with educators. Participants were encouraged to share their successes to encourage comparison within the group. In addition, role models were featured in the case study video
**Credible sources** *(Socially focused to Persuade and Enable)*	Videos were used as part of the intervention including advice and tips from community leaders, healthcare practitioners and patients from the community that have successfully changed their habits
**Feedback on outcomes**,** self-monitoring of behaviour**	The programme started with personal measurements and blood results, and updated outcome measures were given at the end of the programme. Participants were encouraged to monitor weight loss progress by taking waist measurements through the course and completing their programme booklets.
**Self-monitoring of behaviour**,** action planning**	Participants were given pedometers to measure their steps and were taught to develop action plans and measure their progress against them.
**Goal setting** (behaviour)	Participants were guided through setting their own goals for the lifestyle targets that are important for them
**Problem solving**	The *‘task card’* homework activities were discussed at the beginning of each session, challenges were identified, and the group problem solve collectively. Problem solving also forms part of the education sessions about lifestyle habits.
**Action planning**	Participants were guided through how to develop and adjust action plans for each of the target behaviours and for their personal objectives, to help keep them motivated.

## Methods

### Design

Focus groups and semi-structured interviews were conducted as part of a process evaluation within the HEAL-D feasibility randomised controlled trial (RCT). Ethical approval was granted by the NHS Health Research Agency (HRA) registration number 233,419; participants provided written informed consent prior to participation.

### Participants

Participants were taking part in the HEAL-D feasibility trial, a two-arm parallel group RCT; eligible trial participants were adults (age ≥ 18 years), of self-declared Black-African, Black-Caribbean or Black-British ethnicity living with T2D, able to communicate in English, and with no complex diet or learning needs that made group-based education inappropriate. Exclusion criteria included pregnancy and complex clinical needs. Eligibility was confirmed by the participants’ primary care practice or diabetes team [16]. Participants were recruited to the RCT through a combination of community-situated initiatives, such as awareness building in faith institutions and with charity partners, and via primary and secondary care diabetes self-management education referrals. Community recruitment strategies were particularly important as the target communities are often under-represented in medical research and distrust of healthcare services has been reported, particularly amongst those with lower health literacy [23]. All participants allocated to the HEAL-D intervention arm (*n* = 45) were eligible to take part in the process evaluation focus groups. To explore perceptions in greater depth, one to one interviews were then conducted with a purposive sample of participants, to a give a balance of gender and ethnic background. Participation in the focus groups and interviews was optional.

### Procedures

Full details of the HEAL-D intervention have been previously published [22]; in brief, HEAL-D is a group-based programme delivering a curriculum of diet and lifestyle behaviour change support in seven 2-hour sessions, facilitated by a specialist dietitian, a lay educator and exercise trainers. The details of the BCTs identified during the HEAL-D intervention co-design process and how these were operationalised across the intervention components are further illustrated in Table 1. For the process evaluation, collection of the qualitative data was undertaken by an experienced White British doctoral qualitative researcher (AM), who was known to the participants through their involvement in the feasibility trial. For both the focus groups and interviews, participants were briefed that the sessions were being conducted to help the research team understand what worked well and what needed to be improved in the HEAL-D intervention. Sessions were audio recorded and transcribed. Where needed, ethnically concordant transcribers were used to ensure accents were faithfully transcribed and the audio recordings were checked against the transcripts by the research team to ensure accuracy. The topic guides were loosely structured around the COM-B model, exploring changes in behaviour and shifts in capabilities, opportunities and motivations.

One focus group was planned at the end of each seven-session delivery of the HEAL-D programme, thus participants had attended the same group. In some circumstances participants from two programme groups were merged to give sufficient numbers for a focus group. In most cases, the focus group was conducted within two weeks of the intervention finishing. Each focus group lasted approximately 2 h and participants were given a £10 gift voucher for their participation. The focus group was structured using a topic guide which explored experiences of the intervention, key points of learning and impact, and behaviour change. Sessions began with participants describing their response to the intervention and any changes to their health behaviours resulting from their experience. Participants were given the opportunity for non-judgmental recall to understand the key drivers of change for each individual, followed by further probing around specific components.

The semi-structured interviews were conducted to gain further insight into the focus group discussions, as well as to explore personal perspectives. The interviews were conducted in person in university rooms, the participant’s home or within a medical setting, depending on participant preference, and lasted approximately 60 min. They took place within 4 weeks of the intervention finishing, with participants being given a £10 voucher for their time [23]. The interviews followed a similar topic guide to the focus groups but gave the opportunity to probe topics in more detail (see supplementary file 2 for more detail on the topic guide). Certain sections of the topic guide were iteratively developed as the focus groups were conducted, allowing further exploration of areas needing clarification. Additionally, it enabled the participants to share their perspective on more sensitive topics p, such as the interaction between people from different ethnic backgrounds and between genders.

### Analysis

A deductive approach was taken in the analysis [24]; data were coded using a framework approach [25], using the BCTs identified in the theory of change developed for the HEAL-D intervention [18] as the basis for categorising the data by interview/focus group. General comments on the intervention as well as descriptions of behaviour change were also recorded. This approach enabled us to understand the impact of each BCT used in the intervention and facilitated an intersectional exploration of the influence of contextual factors on the way the BCTs supported behaviour change e.g., age, gender, ethnicity. BCTs were considered to be effective when participants described the associated intervention components as being valuable to them achieving behavioural changes or shifts in their knowledge, capability or motivation. To give a general indication of the BCTs considered most beneficial by the participants, the interview transcripts were coded for recall of positive impacts of each BCT by participants and results recorded in a coding density chart. The focus group data were not included in the coding density chart due to the challenge of accurately matching the verbatim quotes to individuals. NVivo software was used to facilitate the analysis. The coding was carried out by one researcher (AM) and reviewed by the study Chief Investigator (LG) and the qualitative lead (CR) to ensure agreement across the team. This peer auditing approach is recommended to ensure credibility [26, 27]. The data analysis was shared with participant advisors to inform and confirm the qualitative analyses. In addition, interviews were held with two educators delivering the intervention to understand their perspective on how the intervention worked in practice (which BCTS and components achieved engagement).

The data are reported according to the COREQ guidelines (supplementary data 1).

## Results

Thirty-six participants took part in this study (80% of those eligible); 15 participated in interview only, 16 in focus group only, and 5 took part in both an interview and focus group, Table 2. Four focus groups were conducted with 4 to 7 participants in each. The sample was 83% first generation, predominantly female, with both Black African and Caribbean ethnicities represented.

**Table 2:** Participant characteristics

	% (*n*)	Mean (SD)
Ethnicity		
Black African	44.4 (16)	
Black Caribbean	50.0 (18)	
Mixed race (BA/WB)	5.6 (2)	
Gender		
Female	61.1 (22)	
Age		59.5 (10.02)
Migration history		
First generation	83.3 (30)	
Second generation	16.7 (6)	

**Fig. 1:**
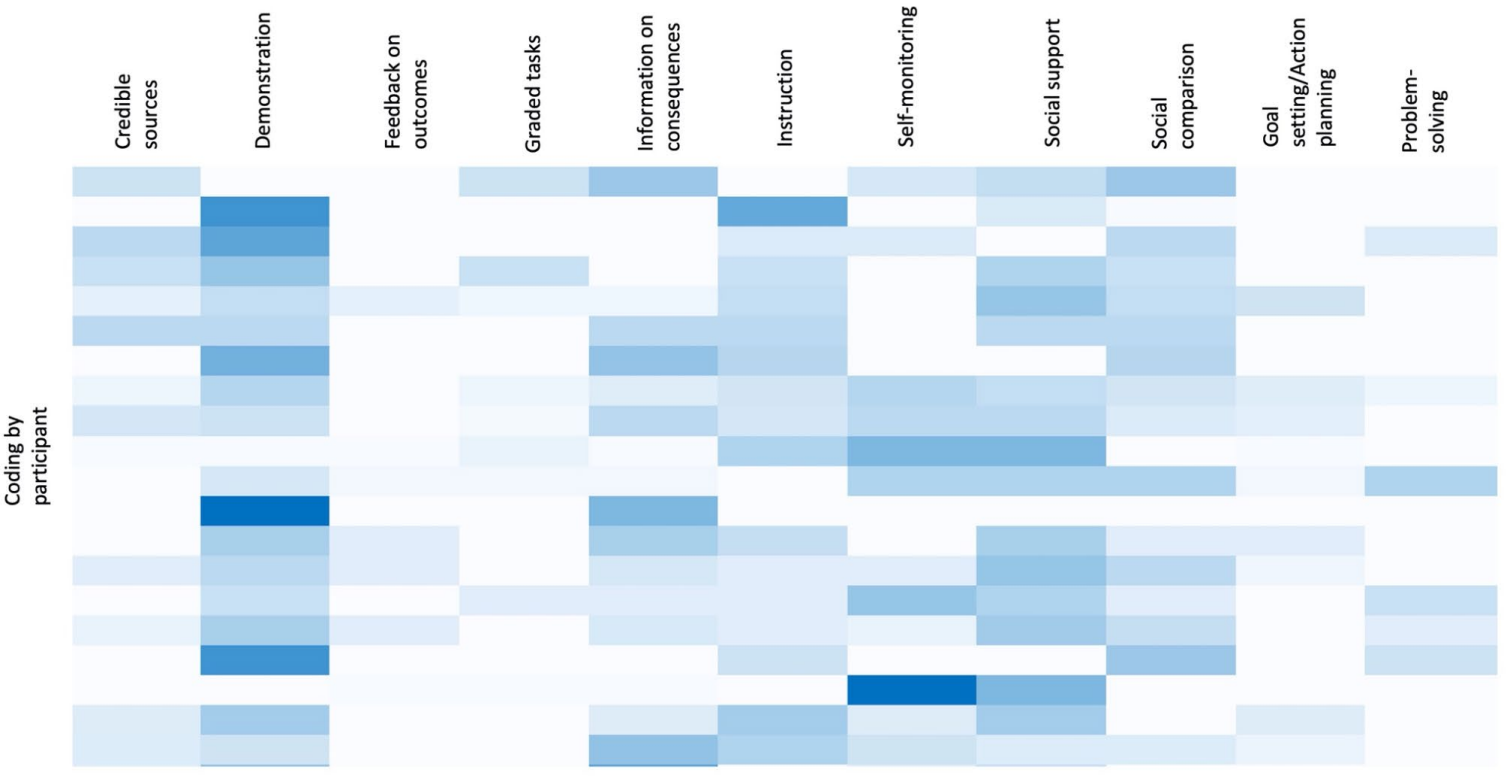
Coding density by BCT by interview participant (the darker blue indicates greater coding density). Each line corresponds to one participant

Overall, participants reported positive changes in their self-management behaviour as a result of participating in the HEAL-D intervention. Full details of the differences in baseline and endpoint biomarkers and anthropometry are reported elsewhere [17]; here we refer to self-reported behaviour change. Figure 1 illustrates the components that were most commonly reported to have supported behaviour change. Principally, these were BCTs to *demonstrate the behaviour*, which align to the educate function; those based on providing *social support* and *social comparison*, which align to the persuade and enable functions; and those promoting advice and support from *credible sources*, which align to the persuade function.

### Reported behaviour change

The most commonly reported behavioural changes were increased moderate to vigorous physical activity, reduced carbohydrate portions, and engagement in weight-monitoring behaviour; illustrative quotes are provided in Table 3. The majority of participants reported improvements in health such as improved metabolic parameters, weight-loss, improved stamina, and medication reduction. With regards to carbohydrate portions, knowing how portion guidance related to culture-specific starchy foods was empowering, so cultural foods could be enjoyed with confidence: *“You’re armed*,* you’re tooled with the information now.”*^1^ Relatedly, participants reported deciding to decrease or avoid cultural starches for diabetes management, thus changing longstanding eating patterns [17].

**Table 3:** Participant reports of behaviour and self-efficacy changes

*Reduction in starchy carbohydrate intake*	*“Do you know, since the programme, as of the middle of the programme and since the programme ended I have not taken the garri, the cassava one, I’ve not taken it.* *Interviewer: Why is that?* *Because I’ve come to realise after eating that garri, that cassava one, swallow, especially in the evening, I get so heavy in me. I can’t do anything. I was tempted all throughout in Nigeria, but I avoided it. I realised one of the things I avoided was the garri, and I could eat a big bowl of rice… I cut my rice into half now.”* **(Participant 44, BA Male, Age 64, Nigeria)**
Reduction in necessary medication	*“They have taken me off glazi*,* what do you call it? [Glicazide]*,* they’ve taken me off that they said because my blood sugar has gone down*,* I’m okay. I really love it. I’m just left with metformin now.”****(Participant 24***,*** BA Female***,*** Age 58***,*** UK)***
Reduction in waist size	*“I’ve lost weight*,* I think I have. Well*,* it’s funny*,* I still weigh the same*,* but my trouser size is - I was taking size 36 trousers*,* yes? Now*,* all of a sudden*,* they’re too big for me and I’ve started taking size 34 now… Yes*,* it’s inches*,* isn’t it.”****(Participant 58***,*** BC Male***,*** Age 60***,*** Dominica)***
Increased exercise and improved stamina	*“… because of the exercise*,* I mean I’m normally doing bits*,* but I can say that my stamina has increased. For instance*,* I went to Brixton today*,* and months back - I have a bad knee and a hernia - and I found myself walking around Brixton*,* so I maybe did about 45 min walk*,* so my stamina is definitely [improved]. I’m thinking it is nothing to climb up a hill and whatever*,* without puffing and things […] I can feel definitely that my stamina has increased.”****(Participant 66***,*** BC Female***,*** Age 68***,*** Jamaica)***

### BCTs to educate and improve knowledge and skills

The majority of study respondents reported that intervention components based on BCTs to improve knowledge and skills were pivotal to their success; illustrative quotes are presented in Table 4. Participants valued the balance between the theoretical and practical components designed to *demonstrate the behaviour*. In HEAL-D, components based on these BCTs included food related games, participatory physical activity sessions, participatory cook and taste session, alongside verbal and visual information. The information and demonstration techniques focused on cultural traditions with regards to reducing carbohydrate intake, changing cooking habits and engaging in moderate to vigorous physical activity likely successfully facilitated participants’ behaviour change. Components based on other BCTs to increase knowledge worked synergistically with the practical participatory demonstration e.g., the BCT *Information on health consequences* was the basis of a culturally aligned animation video called *Diabetes Explained*, and written, visual and verbal information about the links between behaviour and diabetes outcomes; and *Instruction on how to perform behaviour* was the basis of photographic representations of portion sizes, written details about understanding exercise intensity, and verbal instructions about which foods contain healthy fats. Written resources providing *Instruction* and *Information on health consequences* were valued, with participants viewing them as a reference guide which could be reviewed outside the sessions:^2^ Notably, it was the cultural salience of the information and demonstration techniques that made the difference, compared to information they had received previously. The provision of written and visual materials was supported with the physical presence of the educator who helped reinforce the information. Having an educator there in person was particularly important for older participants who may struggle with biomedical understanding.

**Table 4:** Participant reflections on BCTs chosen to increase knowledge and skills

**BCT**: demonstration; instruction **Component**: Food knowledge game	*“That was the day I realised probably I had been a big fool. […] It was all fun and I took home a lot. It looks like a little game, but it brought the reality, the essence of the food we eat. It brought it into real-life play, and it was quite a very useful method of passing the message, other than just saying it with your mouth.… the shock is not the same thing.”* *** (Participant 44, BA Male, Age 64, Nigeria)***
**BCT**: demonstration; instruction **Component**: Participatory exercise classes	*“There are lots of people*,* you tell them to exercise*,* and they say*,* ‘Oh yes*,* I’ll do it’*,* yes*,* but to actually participate - because I’m sure there were lots of people in that group who never did what we did during that course. They say*,* ‘Oh yes*,* I did*,* I do walking’*,* but it opened your awareness to certain forms of exercise that you need to do.”****(Participant 51***,*** BC Male***,*** Age 77***,*** Guyana)***
**BCT**: demonstration; instruction **Component**: Cook and taste session	*“The food they prepared for us has given me a lot of ideas. I know I have to eat veg*,* but there are different ways you cook veg which they showed us the other day*,* which is very*,* very educative. They teach you how to put little bit of oil*,* not like the way we put. You think about eating veg*,* you put plenty of oil*,* you’ve spoilt it. They taught us a lot*,* that cooking.”****(Participant 34***,*** BA Female***,*** Age 61***,*** Nigeria)***
**BCT**: information about health consequences **Component**: Video	*“The videos are very good. When you are first diagnosed there are a lot of conspiracy theories about what diabetes is*,* or what it’s not. That video tried to explain*,* when you say somebody’s diabetic*,* what is really happening in the inside. It talks about the key*,* which is insulin*,* that will boost the vein*,* and it talks about what happen when the veins is clogged […] So that video is very concise*,* and it explained in the plain language that I think everybody was able to understand.”****(Participant 54***,*** BC Female***,*** Age 55***,*** UK)***
**BCT**: information about health consequences; Instruction **Component**: Written information	*“The book is good because basically*,* what I’ve been doing is going back to my book all the time*,* and that’s another thing its inspiring– you look back at what you’ve learnt. I actually find what works for me is sitting down*,* reading my book– I wrote little things in like if I’d done exercise– you can sit down and write your own experience.”****(Participant 54***,*** BC Female***,*** Age 55***,*** UK)***
**BCT**: information about health consequences; Instruction **Component**: Written information	*“Where we come from*,* your husband can go for another woman*,* just because of food. Like*,* my husband*,* when he’s cooking*,* it’s salt*,* salt*,* salt. He likes salt. So*,* I said to him*,* ‘If you want to cook salt and kill yourself*,* I’ll come to your burial*,* but please don’t add it too much on my one. For me if I had not seen the information I wouldn’t know. I would keep continue doing what we are doing.”****(Participant 64***,*** BA Female***,*** Age 52***,*** Nigeria)***

*“Any problem*,* they are willing to answer our questions if we have anything. She demonstrate things*,* she gets to the board. She tells you what you didn’t know or didn’t understand…The clog. When the blood vessels*,* when they clog. That was like*,* woo! Yes*,* she was explaining over and over how it works. Explained everything. If you had a problem*,* if there’s something that you didn’t understand*,* nothing was too much for them to say*,* ‘Right*,* I can go over it*,*’ or you could stay back and talk to them”* (Participant 62, BC Female, Age 84, Jamaica).

### Socially-focused BCTs to persuade and enable

Intervention components based on *social support* (group sessions facilitating interaction and support) and *social comparison* (e.g., sharing experiences and learning within the group) provided individuals with much needed social support.

*“It made you feel like*,* actually*,* I’m not too - you know*,* you are human because people make errors in assuming that you’re in the same boat as well. So*,* it didn’t make you feel*,* oh*,* you know what*,* you’re so bad at doing this. It just shows you*,* seeing others actually*,* especially if you’re new*,* like me*,* I’m new to it*,* so you’re still finding your feet*,* and it’s okay to fall*,* but just remember*,* you need to pick yourself back up. You can fall*,* but don’t stay down. Pick yourself back up and go straight ahead.”* (Participant 63, BC Female, Age 67, Guyana).

The importance of the group interaction was evident, as participants learned from each other and motivated each other (Table 5). Furthermore, learning from each other gave the educational recommendations credibility and salience, as participants discussed positive personal experiences and insights.

**Table 5:** Participant reflections on socially focused BCTs to persuade and enable

**BCT**: social comparison **Component**: Group sessions	*“That was very good because people don’t really realise that there are people, other people in the same boat as you are, suffering the same thing. Exchanging ideas and views, in some small way, although it might look insignificant, could be helpful to you. By listening to this person or seeing what this person does or hear what this person does, you can benefit from it”* *** (Participant 51, BC Male, Age 77, Guyana)***
**BCT**: social support **Component**: Group sessions	*“If we can share*,* perhaps*,* they were going through a bad time and they’ve relapsed and they’re not sticking to the programme*,* and their diabetes is going out of control. Be able to talk about it and properly try and get them back on track and that kind of thing…”****(Participant 47***,*** BC Female***,*** Age 58***,*** Guyana)***
**BCT**: social support **Component**: Exercise classes	*“Last week I was*,* two weeks ago I was ill. Pains all over my body. So*,* this young lady*,* I told her that*,* ‘I can’t do any exercise today because I’m…’ She came to me*,* face to face and said*,* ‘You can do it’. Said*,* ‘But if you can’t*,* sit down’. So*,* I sat down there. I felt guilty. People are doing*,* I’m not doing. So*,* I start on the chair. When she says*,* ‘Do this’*,* she said*,* ‘Yes. That’s what you should do. You don’t have to stand up*,* you know’. I was feeling the pain but I enjoyed doing the thing. By the time we were finished*,* I stood up. [Laughs] the group…they encourage [me]”****(Participant 65***,*** BA Female***,*** Age 58***,*** Nigeria)***
**BCT**: credible sources **Component**: case study videos	*“The videos about the people that are living with diabetes*,* you see stories about people that encourage you. That some people have been doing this*,* have been diagnosed for years*,* and they are still living happily. That you can still live a happy life*,* even if you are diabetic […] Talking about the food and what we can eat*,* and our food*,* the type of food we have and how much carb there… It was amazing that we didn’t… I still think up to this day a lot of Caribbean*,* African backgrounds*,* we’re still not aware of that*,* and we still eat so much of it. So again*,* it was awareness and I find it was really good.”****(Participant 22***,*** BA Male***,*** Age 41***,*** Nigeria)***

*“I find myself… that I was the least-informed in the group. So*,* I was learning from these elderly ladies and gentlemen. Seriously*,* I was listening to their experience*,* their practical experience and what they thought […] he*,* my fellow countryman… he taught me a lot of things that could really increase diabetes*,* if I was to eat or drink certain things. What I’m saying is that I wasn’t aware before… and it’s like… it was really verifiable information. So*,* it comes back right into say*,* well*,* awareness and discussing and sharing.”* (Participant 22, BA Male, Age 41, Nigeria).

These components supported the learning by improving confidence, making it acceptable to challenge traditions, and normalising diabetes, encouraging individuals to be open about their condition, where they would traditionally have been more hesitant about disclosure.

*“Where I come from*,* from the Northern part of Nigeria*,* it is a shame thing. That shame*,* that*,* ‘Oh*,* how can you tell your family you have this?’ So*,* you keep it to yourself*,* and it’s like AIDS/HIV. So*,* you don’t want to tell no one*,* and just so that you just can continue with it yourself. So*,* it has really helped me broaden out the kind of person I am. Going to these open courses*,* it give you a full mind like to expand your talk and get more confidence. then I thought*,* ‘Oh*,* I can talk now*,* too”* (Participant 64, BA Female, Age 52, Nigeria).

Furthermore, it created strength to resist social pressures. The use of *credible sources* and *modelling* e.g., having ethnically concordant lay educators and videos with tips from faith leaders and other trusted members of local Black communities, reinforced the social acceptability of the new behaviours.

*“The videos about the people that are living with diabetes*,* you see stories about people that encourage you. That some people have been doing this*,* have been diagnosed for years*,* and they are still living happily. That you can still live a happy life*,* even if you are diabetic […] Talking about the food and what we can eat*,* and our food*,* the type of food we have and how much carb there… It was amazing that we didn’t… I still think up to this day a lot of Caribbean*,* African backgrounds*,* we’re still not aware of that*,* and we still eat so much of it. So again*,* it was awareness and I find it was really good.”* (Participant 22, BA Male, Age 41, Nigeria).

### BCTs to improve self-efficacy and behaviour regulation

Components associated with BCTs to improve self-efficacy and behaviour regulation included *self-monitoring* through measuring waist, weekly weighing, and using a pedometer; *goal setting* and *action planning*^3^ through a SMART goal session and exercises, and weekly group goals; *Feedback on outcomes* through anthropometry and biochemical markers at the end of the intervention; and *Problem solving*. In general, these BCTs were less frequently mentioned than the components associated with improving knowledge and developing social support. Yet, they still seemed powerful for several individuals (Table 6) with many participants reporting setting themselves goals for the programme, from fitting into a dress that had become too tight to achieving 10,000 steps every day.

**Table 6:** Participant reflections on BCTS to improve self-efficacy and behaviour regulation

**BCT**: Goal setting **Component**: SMART goals	*“I was shopping yesterday and I was walking around Sainsbury’s, and because you know we’ve got that goal target, of getting your waistline down? So, I’m about one inch away from my goal target… All I kept hearing, every time I walked past something naughty was, “one inch away from 33, don’t do it”! We were actively encouraged. Like… ‘This is a challenge, set yourself a goal every week. Set yourself a new different goal,’ which is good because even now, like I said, I’m one step away from my waist goal, and I’m already thinking, all right, when I hit that, I’ve got to do the next goal. So, I’m always planning that in my head already.”* *** (Participant 61, BC Female, Age 37, UK)***
**BCT**: Feedback on outcomes **Component**: HbA1C measurement	*“My blood HbAc [sic] when I went to my GP a few weeks ago*,* it was 53. I feel really good. I feel*,* ‘Wow*,* all this hard work is working*,*’ but it’s not just something you do for three months and then think*,* oh*,* I’ll go back to it. It’s an ongoing thing. This is what people have to understand*,* it’s not something you can just switch off for a few months and then think*,* oh*,* I’ll come back to that. It’s literally ongoing all the time.”****(Participant 41***,*** BC Female***,*** Age 58***,*** Barbados)***
**BCT**: Self-monitoring **Component**: weighing and measuring waist	*“Yes*,* I’ve been weighing*,* and I’ve been taking my measurement round*,* you know*,* bust*,* stomach*,* or especially my long waist*,* and I’m wearing dresses that I wasn’t going into. I hate to give away my dresses because they are expensive. Yes. So*,* I appreciate coming to this course*,* and I can see. I used to have segmented neck here*,* but now when I look on the mirror*,* I see that’s reduced. I am feeling better… I can even fight my husband now!”****(Participant 63***,*** BC Female***,*** Age 67***,*** Guyana)***

*“We were actively encouraged. Like*,* ‘This is a challenge*,* set yourself a goal every week. Set yourself a new different goal*,*’ which is good because even now*,* like I said*,* I’m one step away from my waist goal*,* and I’m already thinking*,* all right*,* when I hit that*,* I’ve got to do the next goal. So*,* I’m always planning that in my head already.”* (Participant 60, BA Female, Age 49, Sierra Leone).

Those participants who set themselves a personal goal found it motivating and were assisted by the *Self-monitoring* element facilitated by the pedometer and waist tape measure: *“When I saw the size 34 waist*,* it just gave me a bounce in my step. I was good for the day.”*^4^ When individuals saw their Hba1C, weight and other parameters had reduced it reinforced the changes they had made.

*“The blood sugar has really gone down […] This thing dropped from seven and it’s six. […] I think if you really look at my progress now*,* I think*,* I don’t know how*,* I’m so confident. At the moment*,* I just feel that there’s nothing wrong with me… even though there’s still much to do*,* but I think I’ve really improved a lot in so many ways*,* and so… Just them telling me… I’m just happy… Just I have a good feeling for myself*,* you know*,* very confident*,* very confident. It’s just like there’s nothing disturbing me. It’s just like part and parcel of me now. […] Before all the food*,* my living*,* the way I live now is*,* I don’t know how I can describe it to you*,* the gap is so big.”* (Participant 18, BA Male, Age 42, Cameroon).

## Discussion

The aim of this study was to understand how the HEAL-D intervention supported behaviour change by exploring participant responses to the chosen BCTs. Participants reported that the HEAL-D intervention helped them change their diabetes-related health behaviours, particularly in relation to increasing moderate to vigorous physical activity, reducing carbohydrate portion size, and engaging in behaviours to manage weight. This suggests that the selected BCTs were acceptable and perceived to be effective and have practical utility in a real-world setting. Those which had most impact were *demonstration of the behaviour*,* social support*,* social comparison and credible sources.* Of the other BCTs embedded in the HEAL-D intervention, those felt to be necessary but of secondary importance were BCTs to educate (*Information about health consequences*,* instruction how to perform the behaviour*) and to improve self-efficacy and behaviour-regulation (*Self-monitoring*). Less impactful BCTs were *problem solving*,* graded tasks*,* goal setting and feedback on outcomes.*

People from minority ethnic backgrounds living with T2D in high income countries often experience conflict between diabetes-related lifestyle guidance and social norms, particularly normative diet traditions, body image ideals and beliefs around purposeful physical activity [28, 29]. The strong reliance on starchy staples as well as oil and salt in cooking means that some dietary change is warranted to support diabetes management, although it does not require cultural foods to be eliminated from the diet. Dietary change is challenging for many individuals as habits are formed early in life [30]. However, for migrant communities cultural foods often play a symbolic role in maintaining cultural identity [31]. The role of food culture is considered crucial in the diasporic experience [32]. It can help delineate ethnicity, creating a sense of “us” [33], maintains a link with homeland [34], and reinforces social bonds [32, 35]. Individual dietary-related behaviour change can be further impeded in African collectivist cultures, where family and group preferences are given precedence over those of the individual [29]. Our analysis highlights the value of BCTs that increase social opportunity (*social comparison*,* credible sources*,* social support*,* modelling*) to alter beliefs about the incompatibility of self-management behaviour change with cultural identity, and, interestingly, to challenge the perceptions of others within their communities. The additional provision of culturally pertinent guidance on portion size (*instruction on the behaviour*,* demonstration*) empowered individuals to make dietary changes while still following cultural preferences. These findings support the development of specific interventions or adaptations for specific cultural groups and suggest the importance of community delivery or involvement in intervention delivery.

This evaluation also highlights the value of experiential learning amongst the participants, particularly components designed to deliver the *demonstration* BCT, such as learning games and cooking sessions. This finding supports much of the existing literature that reports the importance of kinship, social interaction and collectivism for those of Black African ancestry [36–38]. In communities of African ancestry living in high income countries, lack of social support has been identified as a specific barrier to lifestyle-related health behaviour change [39–41], particularly when healthcare advice may conflict with culturally influenced social norms [42]. Lifestyle interventions for African American individuals that focus on engaging in social networks have been shown to be particularly effective [41, 43], and a review of weight management interventions to support African American women suggests that mobilising social support may be both *“therapeutic and cost-effective”*, acting to improve self-efficacy and individual perception of control [41]. Moreover, the presence of social support, such as family members with diabetes, has been shown to be associated with positive diabetes self-management behaviour in African American communities [44].

Notably, some of the BCTs that have their origins in control theory, such as *action planning*, *goal setting*,* self-monitoring*,* feedback on outcomes*, which have been identified in the development of other interventions to support lifestyle change, were less often mentioned in this analysis, although were salient for some. These BCTs have been shown to be important in self-regulation for individuals living with diabetes [45]. In addition, several reviews highlight the value of these techniques in supporting change in physical activity and dietary behaviours [45–47]. Our data suggest that these BCTs were important to include, although appeared not to be central to supporting behaviour change.

This analysis has strengths in the use of rigorous qualitative methods with analysis triangulated with patient and community advisors, an independent qualitative advisor, and community-driven recruitment to improve inclusivity. COM-B has now been used in formative research to identify BCTs and help specify the theory of change in the design of several interventions for diabetes [48–51] and gestational diabetes [52–54], but its application in study populations from minority ethnicities is limited [51, 52, 54]. To our knowledge, none of these studies have reported process evaluations specifically related to the effectiveness and acceptability of identified BCTs in practice. Whilst a substantive multi-centre trial evaluation of the HEAL-D intervention is now in progress, the current evaluation was conducted in London, where there is a high population density of people from minority ethnicities. This may influence transferability to other geographies with a different ethnic density, where community dynamics and healthcare support may differ. The researcher conducting the data collection for this evaluation was also involved in the intervention co-design and development, which had the benefit of increasing rapport and familiarity with participants, supporting frank and open discussion. However, using an independent researcher may have been preferable to prevent any introduction of bias. Triangulation of the analysis within the research team, participant representatives and data from educators delivering the intervention helped minimise bias resulting from this.

## Conclusions

Our evaluation suggests that the COM-B/Behaviour change wheel methodology supported the identification of BCTs that were acceptable and effective in promoting behaviour change in the HEAL-D intervention. BCTs promoting social opportunity appear to be particularly effective in supporting lifestyle behaviour change in Black African and Caribbean adults with T2D, where social norms can conflict with diabetes self-management guidance. Additionally, lifestyle interventions should include opportunity for experiential learning alongside provision of information. These findings may be of value to researchers and clinicians developing health promotion interventions for people of Black African and Caribbean ethnicity, and researchers using COM-B and the Behaviour Change Wheel, as there is little published evaluation of the acceptability and effectiveness of this methodology for selecting BCTs to support behaviour change in people from minority ethnicities.

## Electronic supplementary material

Below is the link to the electronic supplementary material.

Supplementary Material 1

Supplementary Material 2

## Data Availability

The data set from this study is not publicly available due to the restrictions associated with ethical approval but are available upon reasonable request to the corresponding author [LMG].

## Footnotes

1 Participant 60, BA Female, Age, 37, Sierra Leone.

2 Participant 17, BA Male, Age 57, Sierra Leone.

3 It was difficult to distinguish between Action planning and Goal setting in the data so the two BCTs are treated collectively.

4 Participant 61, BC Female, Age 37, UK.

## Abbreviations

BA: Black African
BC: Black Caribbean
BCT: Behaviour change technique
BMI: Body Mass Index
HEAL-D: Healthy Living and Active Lifestyles for Diabetes
MR: Mixed race
NHS: National Health Service
NICE: National Institute for Clinical Excellence
RCT: Randomised controlled trial
SMART goals: Specific, Measurable, Achievable, Relevant, Time-bound goals
T2D: Type 2 diabetes
WB: White British
