# Supplementary material for: A qualitative evaluation of the effectiveness of behaviour change techniques used in the Healthy Eating and Active Lifestyles for Diabetes (HEAL-D) intervention
Source: BMC Public Health. 2025 Feb 11;25:568. doi: 10.1186/s12889-025-21767-8 (PMC11817571; doi:10.1186/s12889-025-21767-8)
Supplement: Supplementary file 2 — Supplementary Material 2 [file 12889_2025_21767_MOESM2_ESM.docx]

**Example interview and focus group questions**

**Feelings about diabetes**

When were you diagnosed with diabetes?
What do you feel about having diabetes?
Have your feelings about diabetes changed since doing the HEAL-D course? How?
Had you attended any other diabetes education course? If so what? How was HEAL-D different?

**Feelings about self-management**

How important do you think it is that a person makes changes themselves in their lifestyle to help manage diabetes?
Did you feel more able to do that after the HEAL-D sessions?
What were the main changes you have made to help the way you manage your diabetes?

- Explore PA before and after HEAL-D
- Weight before and after
- Dietary changes before & after

What were the key things you learnt?
Do you feel you will continue with any changes you have made? What will you find hard?
What are the main barriers that stop people from managing their own diabetes?

**Memories of the HEAL-D programme**

For you what parts of the programme do you remember most?
What were the activities that you found most engaging?
Is there any particular activity or element of the programme that you found particularly impactful? Why was that do you think?

**Capability**

**Knowledge & behavioural regulation**

What were the most useful things you learnt during the HEAL-D programme?

- What did you find useful about the dietary information?
- What did you find useful about the physical activity information?
- What did you find useful about the weight management information?

Was the information covered in enough detail for you? (or too much detail)
What changes did you make to your lifestyle (diet, physical activity as a result of attending the HEAL-D programme?
How has your physical activity levels changed?
What changes to the way your food is cooked at home?

Did you find the action planning and goal-setting parts of the programme made it more likely you achieved your goals? Could you explain?
What did you think of the exercise sessions and HEAL-D videos?

**Opportunity**

**Social**

How supportive are your friends and family of any changes you want to make to help your diabetes?

Where so you get the most support from?

What did you think about having group sessions?

- Why do you think the group sessions worked/did not work for you?
- Did join in any events or stay in contact with any of the members of the group?

Did you bring a friend or family member to the sessions? How did you find that?

What did you find helpful about the group sessions? What would you change?

Did you keep in touch with people after the course? How has that helped you?

**Environmental**

Thinking about physical activity – how does the area you live support your efforts to be active?

Do you think cost is a barrier to managing diabetes? Could you tell me why you say that?

**Motivation**

How confident are you that you will eat healthily in the future to support your diabetes?

What do you think you will find difficult?

How confident are you that you will be physically active in future to support your diabetes management?

What do you feel about your weight now and whether that will change further?

Do you feel your weight is important part of diabetes management?

How do attitudes of your friends and family influence your weight management?

Do you take medication? What do you think about that?

**Practical details about the HEAL-D programme**

What did you think was most useful about the programme?

What would you change?

You were given a resistance band, pedometer and exercise videos… how useful did you find these?

- Did you use the HEAL-D exercise videos?
- Other activity equipment?
- What did you think was the value of having instructors over just telling people to exercise more?

How useful were the dietary information booklets? How could they be improved?

How useful were the work books? What would you change?

Do you remember the videos of other patients telling their stories? Were they helpful? Why?

You did practical games to learn about salt and carbohydrate. How did you find that?

**Attendance**

How convenient was the schedule of classes?

What did you think about the venue?

**Research process**

Have you been involved in research before?

How do you find being part of the research process?

How important was it to you to receive the financial payments that part of the research process?

Would you take part in research again?
